# Hashimoto’s thyroiditis and its activity status influence the assessment of lymph node metastasis of thyroid cancer

**DOI:** 10.3389/fendo.2025.1567181

**Published:** 2025-05-27

**Authors:** Caigu Yan, Yuxuan Zhao, Qingyu Zhang, Xianghui He

**Affiliations:** ^1^ Department of General Surgery, Tianjin Medical University General Hospital, Tianjin, China; ^2^ Department of Gastroenterology, Tianjin Medical University General Hospital, Tianjin, China

**Keywords:** papillary thyroid carcinoma, lymph nodes metastasis, Hashimoto’s, immune infiltration, sonography

## Abstract

**Background and purpose:**

Hashimoto’s thyroiditis plays a crucial role in the biological behavior of papillary thyroid carcinoma. The purpose of this study was to explore the impact of Hashimoto’s thyroiditis on the preoperative evaluation of thyroid cancer.

**Method:**

Univariate and multivariate analyses were performed to explore the clinicopathological characteristics and the risk factors for lymph node metastasis (LNM) in 2,261 patients with papillary thyroid carcinoma.

**Results:**

The clinical data showed that the clinicopathological characteristics varied in different states of Hashimoto’s thyroiditis and levels of the thyroid peroxidase (TPO) antibody (*p* < 0.05). In cases without Hashimoto’s thyroiditis, the multivariate analysis showed that male sex (OR = 1.991, 95%CI = 1.574–2.517, *p* < 0.05) was the independent risk factor for LNM, but not in the cases with concurrent Hashimoto’s thyroiditis. The area under the receiver operating characteristic (ROC) curve of the non-Hashimoto’s thyroiditis cases was 0.727 (95% CI = 0.703–0.752, *p* < 0.05), while that in cases with Hashimoto’s thyroiditis was 0.632 (95% CI = 0.590–0.674, *p* < 0.05). Analysis of the differentially expressed genes in the different subgroups found that, in men, the differential genes among the different LNM statuses were mainly enriched in immune pathways, while in women and in younger patients, the genes were mainly enriched in cytokine and kinase pathways; in older patients, the genes were enriched in the extracellular matrix.

**Conclusion:**

Hashimoto’s thyroiditis can affect the preoperative evaluation of thyroid cancer. In addition, sex might affect the biological behavior of papillary thyroid carcinoma, which may result from the different immune and cellular statuses among different sexes and ages.

## 1 Introduction

Papillary thyroid carcinoma (PTC) has become one of the most common malignant endocrine tumors, the incidence of which has increased in recent years (1, 2). At the same time, there is an increasing prevalence of Hashimoto’s thyroiditis, particularly in the youth, which is considered to be closely related to a disordered circadian rhythm (3, 4). Hashimoto’s thyroiditis is considered to be the etiology of thyroid cancer for immune-related factors (5, 6). Moreover, studies have reported that Hashimoto’s thyroiditis appears to perform a dimmorphic role in the biological behavior of PTC in that it leads to tumorigenesis while resulting in a better prognosis (7, 8). Due to the concurrence of Hashimoto’s thyroiditis, the boundary and the calcification of thyroid cancer may be misjudged under sonography (9). PTC has a high rate of lymph node metastasis (LNM), which is an aggressive marker and a prognostic indicator of the tumor. There have been numerous studies assessing the risk factors for LNM (10–12), with the conclusions being varied with a low prediction efficiency, which may have resulted from the confounded screening of participants. Our previous study found that Hashimoto’s thyroiditis and female sex were protective factors for LNM (13) and, as a consensus, that Hashimoto’s thyroiditis was biased toward the female sex (3). For these reasons, we speculated whether the influence of sex in thyroid cancer is caused by immune infiltration.

The aim of this single-center study was to analyze the impact of Hashimoto’s thyroiditis and its activity status on the assessment of LNM in order to further explore the association between sex/immune-related factors and the biological behavior of thyroid cancer.

## 2 Materials and methods

### 2.1 Clinical data

**Figure 1:**
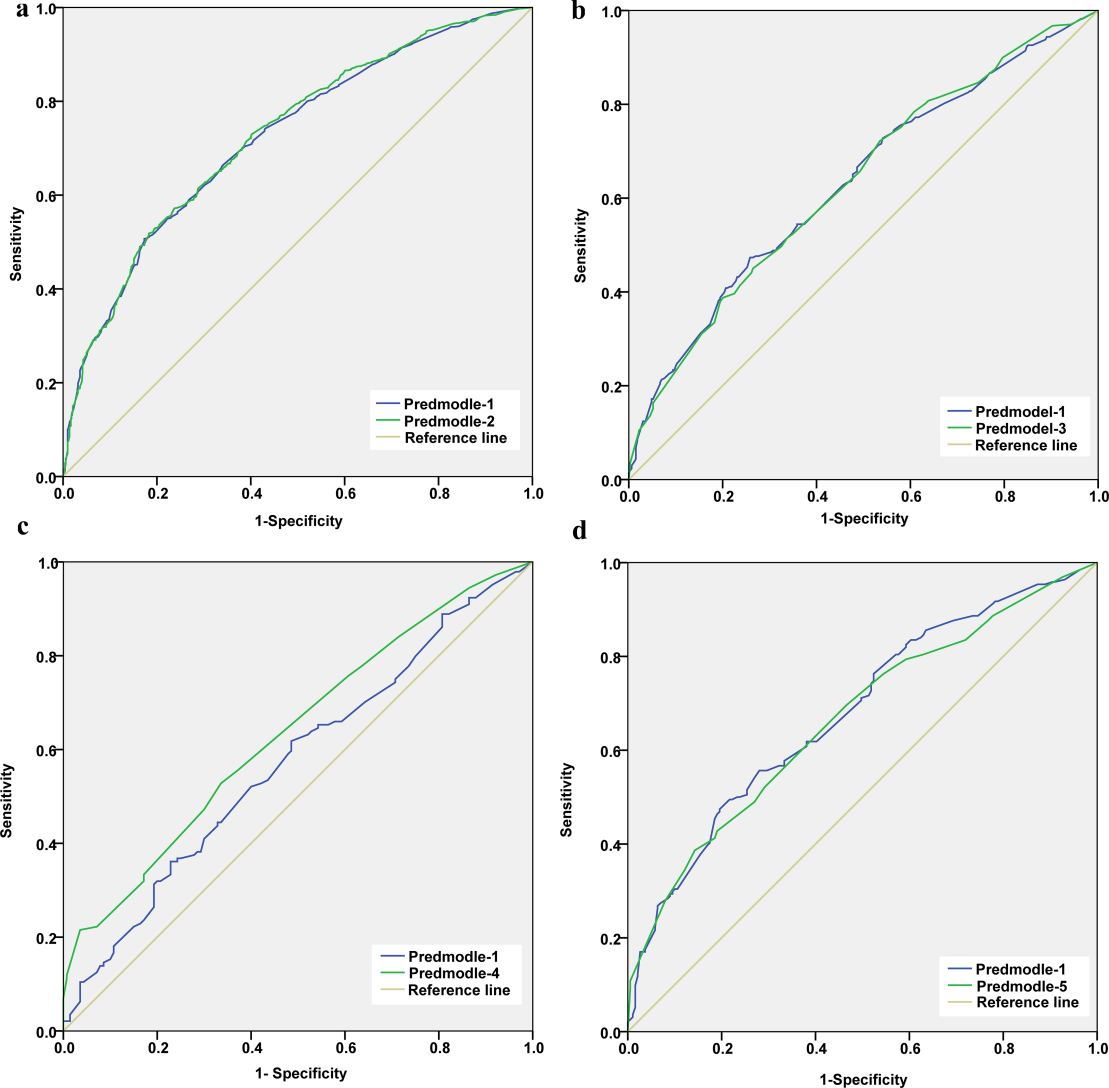
**(a, b)** Receiver operating characteristic (ROC) curves of the lymph node metastasis (LNM) prediction model in non-Hashimoto’s thyroiditis cases **(a)** and in cases with Hashimoto’s thyroiditis **(b)**. **(c, d)** ROC curves of the LNM prediction model in cases with normal thyroid peroxidase (TPO) antibody **(c)** and in those with high TPO antibody **(d)**.

A total of 2,261 patients from 2014 to 2023 were selected, all of whom were diagnosed with PTC and underwent surgery by the same doctor. In our center, all patients diagnosed with PTC undergo routine prophylactic central compartment lymph node dissection, and the pathological results were obtained separately by two experienced pathologists. The inclusion criteria were: 1) initial PTC surgery and 2) complete clinical and pathological data. The exclusion criteria were: 1) recurrent PTC and 2) incidental PTC without central lymph node dissection (**Figure 1**).

The cutoff age was set to 45 years, which meets the requirements of the 7th edition of the American Joint Committee on Cancer (AJCC) and conforms to the peak prevalence of autoimmune disease in women. Hashimoto’s thyroiditis was defined as lymphocytic infiltration and the formation of germinal centers combined with an elevated serum thyroid peroxidase (TPO) antibody. The TPO antibody is considered as the marker of the activity status of Hashimoto’s thyroiditis. Based on the clinical characteristics, all participants were divided as follows: aspect ratio (height divided by width, less than 1 or more than 1), margin (clear or unclear), and calcification (absent or present); preoperative serum assay: thyroid-stimulating hormone (TSH; less than or more than 2 μIU/ml), TPO antibody (normal or high, 35 IU/ml), and serum thyroglobulin (less than or more than 40 ng/ml); and postoperative pathology: tumor size (less than 1 cm, 1–2 cm, and more than 2 cm), multifocality (absent or present), bilateral tumor (absent or present), extrathyroidal extension (no capsule contacting, invading capsule, and violating surrounding tissues), Hashimoto’s thyroiditis (absent or present), nodular goiter (absent or present), and LNM (absent or present).

### 2.2 Public data of gene expression

We downloaded the gene expression data and clinical data of PTC from The Cancer Genome Atlas (TCGA) database (University of California, Santa Cruz; https://xenabrowser.net/) and excluded those cases without LNM information. Furthermore, 497 cases with complete age and sex information were selected, and the included genes were normally determined in at least 75% of the participants.

### 2.2 Statistical analysis

SPSS 22.0 software was used for analysis of the statistical data. Continuous measurement data were expressed as $\bar{x}$ ± *S*, and univariate logistic regression analysis or the chi-square test was performed for single-factor analysis. Multivariate analysis was performed with multivariate logistic regression analysis. A *p*-value <0.05 indicates a statistically significant difference.

## 3 Results

### 3.1 Clinical data and single-factor analysis of LNM

According to the clinicopathological characteristics of the 2,261 cases, 667 patients had concurrent Hashimoto’s thyroiditis and 1,594 without. The average age of the patients without Hashimoto’s thyroiditis was 45.6 years, while that of patients with Hashimoto’s thyroiditis was 43.3 years (*p* < 0.05). Moreover, women comprised a higher proportion of patients with Hashimoto’s thyroiditis than those without, who had lower serum levels of TSH, thyroglobulin, and TPO antibody (*p* < 0.05). Moreover, patients with Hashimoto’s thyroiditis were more likely to have a smaller size (*p* < 0.05) and a lower probability of LNM; however, the difference was not significant (**Table 1**). In the analysis of patients with different Hashimoto’s thyroiditis statuses, it was found that patients with higher TPO antibody levels were younger and had higher TSH levels (*p* < 0.05). Moreover, the size of the tumor and the concurrent calcification varied in different levels of the TPO antibody; however, this did not affect LNM (**Table 2**).

**Table 1:** Clinicopathological characteristics of papillary thyroid carcinoma (PTC) in the 2,261 patients.

Parameter	Without HT (*n* = 1,594)		With HT (*n* = 667)		*p*
	*n*	Rate (%)	*n*	Rate (%)	
Age (years)	45.6 ± ± 12.7		43.3 ± ± 12.7		0.001
<45	784	49.2	380	56.9	
≥45	810	50.8	287	43.1	
Sex					0.000
Women	1,066	66.9	596	89.4	
Men	528	33.1	71	10.6	
Aspect ratio					0.000
≤1	719	45.1	240	36.0	
>1	875	54.9	427	64.0	
Calcification					0.757
Absent	409	25.7	167	25.0	
Present	1,185	74.3	500	75.0	
Ultrasonography margin					0.004
Clear	551	34.6	189	28.3	
Unclear	1,043	65.4	478	71.7	
TSH (μIU/ml)	2.23 ± 3.41		2.89 ± 4.53		0.000
≤2	899	56.4	277	41.5	
>2	695	43.6	390	58.5	
Thyroglobulin (ng/ml)					0.000
≤40	1,198	75.2	583	87.4	
>40	396	24.8	84	12.6	
TPO antibody					0.000
Normal	1,388	87.1	284	42.6	
Higher	206	12.9	383	57.4	
Tumor size (cm)					0.035
≤1	730	45.8	330	49.5	
1–2	599	37.6	254	38.1	0.522
>2	265	16.6	83	12.4	0.010
Multifocality					0.002
Absent	1039	65.2	389	58.3	
Present	555	34.8	278	41.7	
Bilateral tumor					0.411
Absent	1,230	77.2	504	75.6	
Present	364	22.8	163	24.4	
ETE					0.467
No capsule contacting	415	26.0	183	27.4	
Invading capsule	893	56.0	378	56.7	0.777
Violating surrounding tissues	286	18.0	106	15.9	0.240
Nodular goiter					0.000
Absent	372	23.3	498	74.7	
Present	1,222	76.7	169	25.3	
LNM					0.080
Absent	722	45.3	329	49.3	
Present	872	54.7	338	50.7	

HT, Hashimoto’s thyroiditis; TSH, thyroid-stimulating hormone; ETE, extra thyroidal extension; LNM, lymph node metastasis.

**Table 2:** Clinicopathological characteristics of papillary thyroid carcinoma (PTC) in the 667 Hashimoto’s thyroiditis (HT) patients.

Parameter	Normal TPO (*n* = 284)		High TPO (*n* = 383)		*p*
	*n*	Rate (%)	*n*	Rate (%)	
Age (years)	44.7 ± 12.5		42.3 ± 12.8		0.018
<45	151	53.2	229	59.8	
≥45	133	46.8	154	40.2	
Sex					0.021
Women	263	92.6	333	86.9	
Men	21	7.4	50	13.1	
Aspect ratio					0.241
≤1	95	33.4	145	37.9	
>1	189	67.6	238	62.1	
Calcification					0.002
Absent	88	31.0	79	20.6	
Present	196	69.0	304	79.4	
Ultrasonography margin					0.261
Clear	74	26.1	115	30.0	
Unclear	210	73.9	268	70.0	
TSH (μ IU/ml)	2.66 ± 5.94		3.05 ± 3.07		0.017
≤2	133	46.8	144	37.6	
>2	151	53.2	239	62.4	
Thyroglobulin (ng/ml)					0.217
≤40	243	85.6	340	88.8	
>40	41	14.4	43	11.2	
Tumor size (cm)					0.027
≤1	155	54.6	175	45.7	
1–2	103	36.3	151	39.4	0.122
>2	26	9.1	57	14.9	0.011
Multifocality					0.828
Absent	167	58.8	222	58.0	
Present	117	41.2	161	42.0	
Bilateral tumor					0.535
Absent	218	76.8	286	74.7	
Present	66	23.2	97	25.3	
ETE					0.717
No capsule contacting	82	28.9	101	26.4	
Invading capsule	156	54.9	222	58.0	0.434
Violating surrounding tissues	46	16.2	60	15.6	0.853
Nodular goiter					0.012
Absent	198	69.7	300	78.3	
Present	86	30.3	83	21.7	
LNM					0.990
Absent	140	49.3	189	49.3	
Present	144	50.7	194	50.6	

TPO, thyroid peroxidase; ETE, extra thyroidal extension; LNM, lymph node metastasis.

### 3.2 Single-factor analysis for LNM

Univariate regression analysis was conducted for the different subgroups to explore the risk factors for LNM. Among all patients, LNM occurred in 1,051 (46.5%) cases, while 1,210 (53.5%) had no LNM. It was found that, in patients without Hashimoto’s thyroiditis, factors such as age, sex, aspect ratio, calcification, unclear margin, serum thyroglobulin, tumor size, multifocality, laterality, and capsule invasion were the risk factors (*p* < 0.05). On the other hand, in patients with concurrent Hashimoto’s thyroiditis, age, calcification, tumor size, laterality, capsule invasion, and the concurrence of nodular goiter were the risk factors (*p* < 0.05) (**Table 3**). Further analysis of the patients with Hashimoto’s thyroiditis with different TPO antibody levels showed that tumor size and concurrent nodular goiter were the risk factors in the group with normal TPO antibody levels (*p* < 0.05). In the subgroup with high TPO antibody, age, calcification, tumor size, laterality, and capsule invasion were the risk factors (*p* < 0.05) (**Table 4**).

**Table 3:** Univariate logistic analysis of the risk factors for lymph node metastasis (LNM) in different sexes.

Parameter	Without HT (*n* = 1,594)			With HT (*n* = 667)			Total
	LNM(+) (*n* = 872)	LNM(−) (*n* = 722)	*p*	LNM(+) (*n* = 338)	LNM(−) (*n* = 329)	*p*	*p*
Age (years)			0.000			0.002	0.000
<45	502	282		212	168		
≥45	370	440		126	161		
Sex			0.000			0.313	0.000
Women	524	542		298	298		
Men	348	180		40	31		
Aspect ratio			0.007			0.130	0.001
≤1	420	299		131	109		
>1	452	423		207	220		
Calcification			0.000			0.009	0.000
Absent	165	244		70	97		
Present	707	478		268	232		
Margin			0.002			0.894	0.009
Clear	272	279		95	94		
Unclear	600	443		243	235		
TSH (μIU/ml)			0.320			0.679	0.714
≤2	482	417		143	134		
>2	390	305		195	195		
Thyroglobulin (ng/ml)			0.000			0.549	0.001
≤40	623	575		298	285		
>40	249	147		40	44		
TPO			0.518			0.990	0.686
Normal	755	633		144	140		
Higher	117	89		194	189		
Tumor size (cm)			0.000			0.000	0.000
≤1	312	418		142	188		
1–2	373	226	0.000	143	111	0.002	0.000
>2	187	78	0.000	53	30	0.001	0.000
Multifocality			0.000			0.336	0.000
Absent	524	505		191	198		
Present	348	207		147	131		
Bilateral tumor			0.000			0.026	0.000
Absent	626	604		243	261		
Present	246	118		95	68		
ETE			0.000			0.025	0.000
No capsule contacting	179	236		77	106		
Capsule invading	499	394	0.000	204	174	0.008	0.000
Violating surrounding tissues	194	92	0.000	57	49	0.055	0.000
Nodular goiter			0.513			0.011	0.010
Present	198	174		238	260		
Absent	674	548		100	69		

TPO, thyroid peroxidase; TSH, thyroid-stimulating hormone; ETE, extra thyroidal extension.

**Table 4:** Univariate logistic analysis of the risk factors of lymph node metastasis (LNM) in different thyroid peroxidase (TPO) statuses.

Parameter	Normal (*n* = 284)			Higher (*n* = 383)			Total
	LNM(+) (*n* = 144)	LNM(−) (*n* = 140)	*p*	LNM(+) (*n* = 194)	LNM(−) (*n* = 189)	*p*	*p*
Age (years)			0.562			0.000	0.002
<45	79	72		133	96		
≥45	65	68		61	93		
Sex			0.873			0.265	0.313
Women	133	130		165	168		
Men	11	10		29	21		
Aspect ratio			0.142			0.454	0.130
≤1	54	41		77	68		
>1	90	99		117	121		
Calcification			0.236			0.012	0.009
Absent	40	48		30	49		
Present	104	92		164	140		
Margin			0.681			0.867	0.894
Clear	36	38		59	56		
Unclear	108	102		135	133		
TSH (μIU/ml)			0.542			0.990	0.679
≤2	70	63		73	71		
>2	74	77		121	118		
Thyroglobulin (ng/ml)			0.455			0.122	0.549
≤40	121	122		177	163		
>40	23	18		17	26		
Tumor size (cm)			0.015			0.003	0.000
≤1	70	85		72	103		
1–2	54	49	0.253	89	62	0.001	0.002
>2	20	6	0.005	33	24	0.029	0.001
Multifocality			0.750			0.123	0.336
Absent	86	81		105	117		
Present	58	59		89	72		
Bilateral tumor			0.476			0.021	0.026
Absent	108	110		135	151		
Present	36	30		59	38		
ETE			0.051			0.008	0.025
No capsule contacting	37	45		40	61		
Capsule invading	89	67	0.081	115	107	0.043	0.008
Violating surrounding tissues	18	28	0.512	39	21	0.002	0.055
Nodular goiter			0.008			0.326	0.011
Present	90	108		148	152		
Absent	54	32		46	37		

TSH, thyroid-stimulating hormone; ETE, extra thyroidal extension.

### 3.3 Multifactor analysis of LNM

Based on the results of the univariate analysis, the factors that may be associated with LNM (*p* < 0.05) were included in the multivariate logistic regression model. In cases without Hashimoto’s thyroiditis, the multivariate analysis showed that a larger size (*p* < 0.05), extrathyroidal extension (*p* < 0.05), male sex (OR = 1.991, 95%CI = 1.574–2.517, *p* < 0.05), younger age (OR = 2.364, 95%CI = 1.898–2.941, *p* < 0.05), calcification (OR = 1.823, 95%CI = 1.421–2.399, *p* < 0.05), laterality (OR = 1.542, 95%CI = 1.096–2.169, *p* < 0.05), multifocality (OR = 1.351, 95%CI = 1.009–1.808, *p* < 0.05), and aspect ratio (OR = 0.790, 95%CI = 0.630–0.990, *p* < 0.05) were independent risk factors. On the other hand, in cases with concurrent Hashimoto’s thyroiditis, a larger size (*p* < 0.05), extrathyroidal extension (*p* < 0.05), younger age (OR = 1.709, 95%CI = 1.242–2.352, *p* < 0.05), and concurrent nodular goiter (OR = 1.622, 95%CI = 1.123–2.341, *p* < 0.05) were independent risk factors. In the subgroup with normal levels of the TPO antibody, a larger size (*p* < 0.05) and concurrent nodular goiter (OR = 1.982, 95%CI = 1.158–3.391, *p* < 0.05) were the risk factors, while in patients with high levels of the TPO antibody, a larger size, extrathyroidal extension (*p* < 0.05), and younger age (OR = 2.183, 95%CI = 1.420–3.356, *p* < 0.05) were the risk factors (*p* < 0.05) (**Table 5**).

**Table 5:** Predictive factors for lymph node metastasis ( LNM) in patients with papillary thyroid carcinoma (PTC) in the multiple logistic regression analysis.

Parameter	Without HT		With HT		Total
	*p*	Adjusted OR (95% CI)	*p*	Adjusted OR (95% CI)	*p*
Sex	0.000	1.991 (1.574–2.517)	–	–	0.000
Age (years)	0.000	2.364 (1.898–2.941)	0.001	1.709 (1.242–2.352)	0.000
Tumor size (cm)	0.000		0.000		0.000
1–2	0.000	1.831 (1.439–2.331)	0.002	1.704 (1.215–2.389)	0.000
>2	0.000	2.455 (1.757–3.430)	0.001	2.345 (1.410–3.898)	0.000
ETE	0.000		0.041		0.000
Invading capsule	0.000	1.733 (1.344–2.236)	0.013	1.590 (1.103–2.292)	0.000
Violating surrounding tissues	0.000	2.642 (1.879–3.715)	0.106	1.504 (0.917–2.467)	0.000
Calcification	0.000	1.823 (1.421–2.339)	–	–	0.000
Bilateral tumor	0.013	1.542 (1.096–2.169)	–	–	0.000
Multifocality	0.043	1.351 (1.009–1.808)	–	–	–
Aspect ratio	0.041	0.790 (0.630–0.990)	–	–	–
Nodular goiter	–	–	0.010	1.622 (1.123–2.341)	
Normal TPO			Higher TPO		
Age	–	–	0.000	2.183 (1.420–3.356)	0.000
Tumor size (cm)	0.026		0.004		0.000
1–2	0.265	1.339 (0.801–2.237)	0.002	2.057 (1.297–3.263)	0.000
>2	0.008	3.766 (1.404–10.103)	0.027	2.022 (1.085–3.768)	0.000
ETE	–		0.006		0.000
Invading capsule	–	–	0.024	1.775 (1.079–2.920)	0.000
Violating surrounding tissues	–	–	0.002	2.938 (1.478–5.841)	0.000
Nodular goiter	0.013	1.982 (1.158–3.391)	–	–	–

TPO, thyroid peroxidase; ETE, extra thyroidal extension.

Based on the results of the multivariate logistic regression analysis models, a receiver operating characteristic (ROC) curve was drawn to examine the predictive efficiency of the models. The results showed that the area under the ROC curve (AUC) of the non-Hashimoto’s thyroiditis cases **A** was 0.727 (95% CI = 0.703–0.752, *p* < 0.05) (**Figure 1a**, line predmodel 2), while that in Hashimoto’s thyroiditis cases was 0.632 (95% CI = 0.590–0.674) (**Figure 1b**, line predmodel 3). In those with normal levels of the TPO antibody, the AUC **C** was 0.636 (95% CI = 0.572–0.699, *p* < 0.05) (**Figure 1c**, line predmodel 4), while in those with higher levels, the AUC was 0.667 (95% CI = 0.613–0.720) (**Figure 1d**, line predmodel 5). For the model based on the overall participants, the AUC showed similar results (**Figures 1a–d**, line predmodel 1).

### 3.4 TCGA analysis of the differentially expressed genes and gene function enrichment

**Figure 2:**
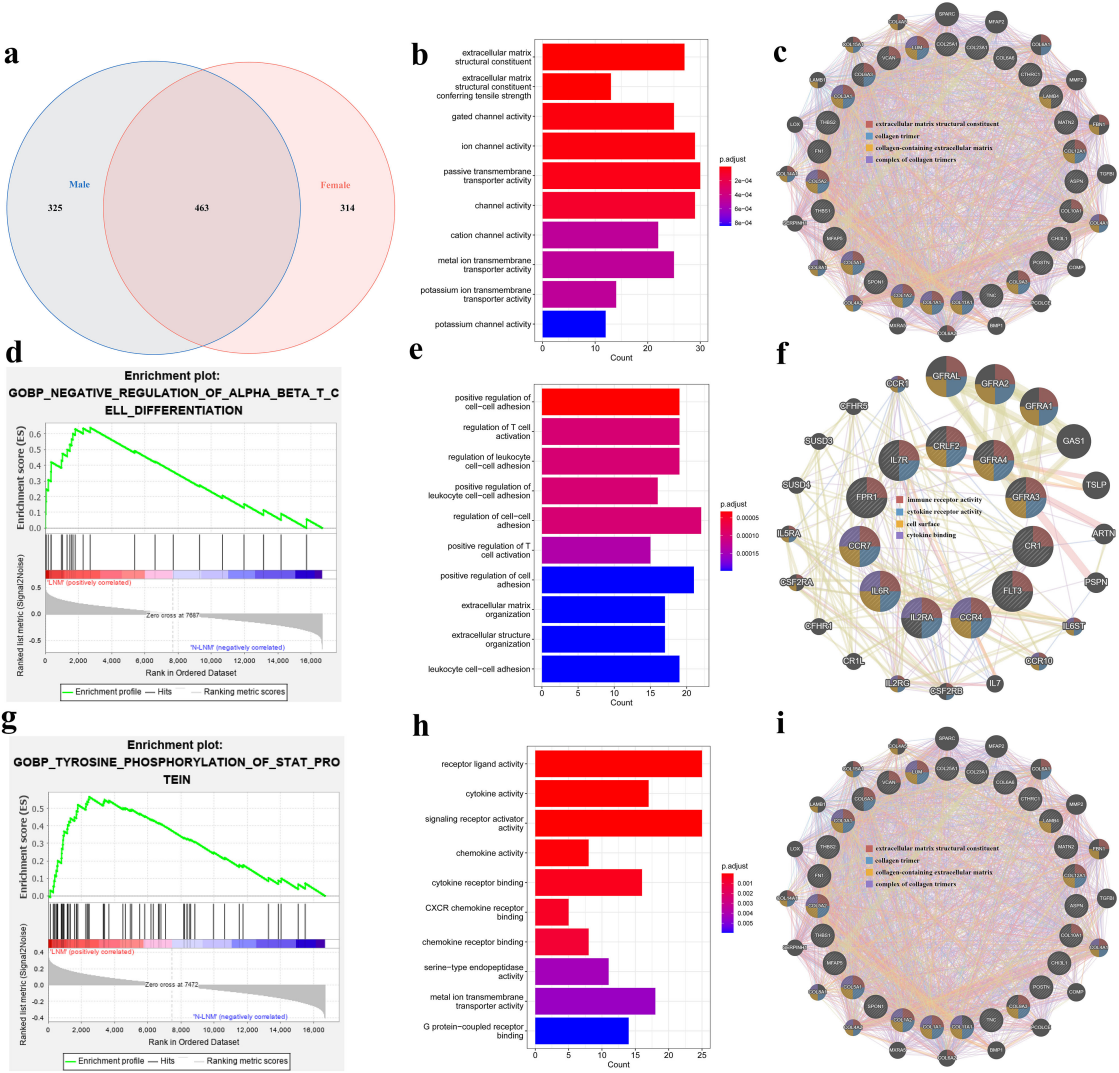
**(a)** Venn diagram of the differentially expressed genes (DEGs) in different lymph node metastasis statuses for the different sexes. **(b)** Gene Ontology (GO) enrichment analysis of the DEGs for the shared genes of the lymph node metastasis status in different sexes. **(c)** Protein–protein interaction networks of the two sexes sharing DEGs for the top one pathway of the GO enrichment analysis in the GENEMANIA database. **(d, g)** Gene set enrichment analysis (GSEA) of the different lymph node metastasis statuses in men **(d)** and in women **(g)**. **(e, h)** GO enrichment analysis of the DEGs unique in men **(e)** and in women **(h)** for the different lymph node metastasis statuses. **(f, i)** Protein–protein interaction networks of the DEGs unique in men **(f)** and in women **(i)** for the top one pathway of the GO enrichment analysis in the GENEMANIA database.

**Figure 3:**
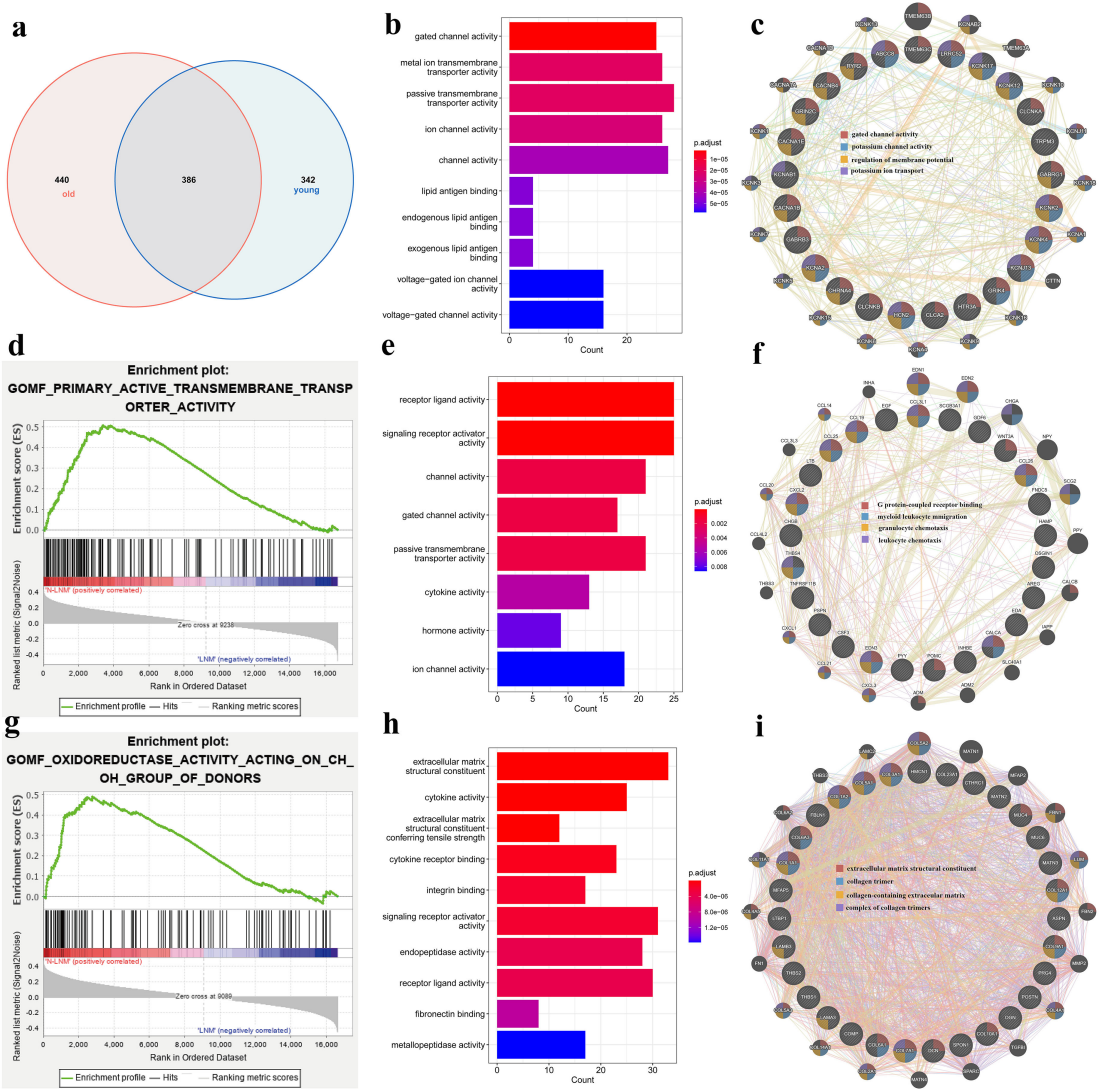
**(a)** Venn diagram of the differentially expressed genes (DEGs) in the different lymph node metastasis statuses for the different ages. **(b)** Gene Ontology ( GO) enrichment analysis of the DEGs for the shared genes of the lymph node metastasis status in different ages. **(c)** Protein–protein interaction networks of the shared DEGs for the top one pathway of the GO enrichment analysis in the GENEMANIA database. **(d, g)** Gene set enrichment analysis (GSEA) of the different lymph node metastasis statuses in younger **(d)** and in older patients **(g)** . **(e, h)** GO enrichment analysis of the DEGs unique in younger **(e)** and in older patients **(h)** for the different lymph node metastasis statuses. **(f, i)** Protein–protein interaction networks of the DEGs unique in younger **(f)** and in older patients **(i)** for the top one pathway of the GO enrichment analysis in the GENEMANIA database.

The gene expression data from TCGA were applied and all subjects were classified into two groups based on sex. Analysis of the differentially expressed genes (DEGs) with different LNM statuses in the two sexes and constituting the Venn diagram by intersecting genes revealed that there were 463 shared genes, with 325 unique in men and 314 unique in women (**Figure 2a**). Enrolling the genes sets for Gene Ontology (GO) enrichment, it was found that the 463 shared genes were mainly enriched in the structural constituent of the extracellular matrix and the transmembrane ion channel, while the genes unique in men were mainly enriched in the immune infiltration pathway and those in women enriched in the cytokine and signaling receptor pathway (**Figures 2b, e, h**), which is consistent with the analysis of the protein–protein interaction networks using the GENEMANIA database (**Figures 3c, f, i**). In the gene set enrichment analysis (GSEA) for the different statuses of LNM, the results showed that the T -cell differentiation pathway was enriched in men and tyrosine phosphorylation enriched in women (*p* < 0.05) (**Figures 2d, h**).

The Venn diagram for the different ages showed that there were 386 shared genes, with 342 unique in younger patients and 440 unique in older patients (**Figure 3a**). The results of GO enrichment showed that the 386 shared genes were mainly enriched in ion transmembrane channel, with the unique genes in the younger group being mainly enriched in the signaling receptor pathway and those in the older group mainly enriched in the extracellular matrix (**Figures 3b, e, h**). The GSEA showed that the DEGs in the younger patients were enriched in transmembrane transporter activity and those in the older patients enriched in oxidoreductase activity (**Figures 3d, g**, p < 0.05). This appears to be a potential mechanism to explain the different LNM patterns in the different populations.

## 4 Discussion

As a self-limiting disease, Hashimoto’s thyroiditis generally manifests in two states: active and resting. During the active state, the immune system attacks the thyroid gland and causes an increased level of the TPO antibody (14). Hashimoto’s thyroiditis might result in hypothyroidism at a later stage and lead to increased TSH levels, then stimulate the proliferation of tumors (15, 16). Our study found that the patients who had concurrent Hashimoto’s thyroiditis were younger, had higher levels of TSH, were smaller in size, and showed a lower possibility of LNM, which is considered as an immune response to the tumor. In general, patients with Hashimoto’s thyroiditis show a diffuse uneven and a hypoechoic sonographic appearance resulting from the infiltration of lymphocytes; at a later stage, they show a calcification change. All of these features may be misjudged as thyroid cancer (17, 18). In our research, we found that, without the background of Hashimoto’s thyroiditis, the parameters evaluated by sonography, such as calcification, aspect ratio, and multifocality, were risk factors for LNM, but were not in cases with Hashimoto’s thyroiditis. In addition, it was found that, in the absence of Hashimoto’s thyroiditis, the model had excellent predictive ability for LNM. However, with concurrent Hashimoto’s thyroiditis, it was difficult to evaluate the possibility of LNM, either in the active or the resting phase. Therefore, we suspect that it is difficult to assess the nodules in the background of Hashimoto’s thyroiditis.

Furthermore, like all autoimmune diseases, Hashimoto’s thyroiditis has a high prevalence in women, which is considered to be a consequence of female sex hormones and the inactivation of chromosome X and fetal microchimerism (19, 20). Other studies have suggested that the immune infiltration and the chronic inflammation caused by Hashimoto’s thyroiditis were the causes of thyroid cancer (21, 22). Our research found that sex was a risk factor for LNM in cases without Hashimoto’s thyroiditis; however, in cases with concurrent Hashimoto’s thyroiditis, sex was no longer a risk factor, either in the active or the resting state. It is not clear whether there is collinearity between these two factors; that is, sex factors affect LNM through the immune status. It has been reported in the literature that the impact of sex-related factors on thyroid cancer sources are from two aspects: one is the alteration of estrogen and estrogen receptors and the other is the varied immune infiltration (23). In addition, the level of thyroglobulin has been used to evaluate recurrence post -surgery. In this study, the level of thyroglobulin in serum was lower in Hashimoto’s thyroiditis, which may be due to the presence of thyroglobulin antibodies (24). The different levels of thyroglobulin can be used to evaluate LNM in cases without Hashimoto’s thyroiditis, but not in those with Hashimoto’s thyroiditis.

Analysis of the differential gene expression in the different LNM states among different genders and age groups revealed different enrichment states. We hypothesized that this difference stems from the fact that patients with thyroid cancer of different sexes and ages show varied hormone levels and immune microenvironments (25–27). This also helps in discovering new methods to prevent and treat PTC in these two aspects in the future.

This study has limitations. It is a single-center study with no external validation; hence, further multicenter studies are required to verify the findings. Furthermore, long-term follow-up is needed to compare the postoperative recurrence risk of pN1 patients with different Hashimoto’s thyroiditis statuses in order to further clarify the impact of Hashimoto’s thyroiditis on tumor recurrence.

## 5 Summary

Hashimoto’s thyroiditis can affect the preoperative evaluation of thyroid cancer. In addition, sex might affect the biological behavior of PTC through immune infiltration.

## Data Availability

The original contributions presented in the study are included in the article/supplementary material. Further inquiries can be directed to the corresponding authors.
